# Astaxanthin Protects Primary Hippocampal Neurons against Noxious Effects of A*β*-Oligomers

**DOI:** 10.1155/2016/3456783

**Published:** 2016-03-01

**Authors:** Pedro Lobos, Barbara Bruna, Alex Cordova, Pablo Barattini, Jose Luis Galáz, Tatiana Adasme, Cecilia Hidalgo, Pablo Muñoz, Andrea Paula-Lima

**Affiliations:** ^1^Biomedical Neuroscience Institute, Faculty of Medicine, University of Chile, 8380453 Santiago, Chile; ^2^Institute of Biomedical Sciences and Center for Molecular Studies of the Cell, Faculty of Medicine, University of Chile, 8380453 Santiago, Chile; ^3^Interdisciplinary Center for Innovation in Health (CIIS), School of Medicine, University of Valparaíso, 2341369 Valparaíso, Chile; ^4^Institute for Research in Dental Sciences, Faculty of Dentistry, University of Chile, 8380492 Santiago, Chile

## Abstract

Increased reactive oxygen species (ROS) generation and the ensuing oxidative stress contribute to Alzheimer's disease pathology. We reported previously that amyloid-*β* peptide oligomers (A*β*Os) produce aberrant Ca^2+^ signals at sublethal concentrations and decrease the expression of type-2 ryanodine receptors (RyR2), which are crucial for hippocampal synaptic plasticity and memory. Here, we investigated whether the antioxidant agent astaxanthin (ATX) protects neurons from A*β*Os-induced excessive mitochondrial ROS generation, NFATc4 activation, and RyR2 mRNA downregulation. To determine mitochondrial H_2_O_2_ production or NFATc4 nuclear translocation, neurons were transfected with plasmids coding for HyperMito or NFATc4-eGFP, respectively. Primary hippocampal cultures were incubated with 0.1 *μ*M ATX for 1.5 h prior to A*β*Os addition (500 nM). We found that incubation with ATX (≤10 *μ*M) for ≤24 h was nontoxic to neurons, evaluated by the live/dead assay. Preincubation with 0.1 *μ*M ATX also prevented the neuronal mitochondrial H_2_O_2_ generation induced within minutes of A*β*Os addition. Longer exposures to A*β*Os (6 h) promoted NFATc4-eGFP nuclear translocation and decreased RyR2 mRNA levels, evaluated by detection of the eGFP-tagged fluorescent plasmid and qPCR, respectively. Preincubation with 0.1 *μ*M ATX prevented both effects. These results indicate that ATX protects neurons from the noxious effects of A*β*Os on mitochondrial ROS production, NFATc4 activation, and RyR2 gene expression downregulation.

## 1. Introduction

Accumulation and aggregation of the amyloid *β* peptide (A*β*) cause neuronal damage and death and induce the cognitive deficits that characterize Alzheimer's disease (AD) [1]. Soluble A*β* oligomers (A*β*Os) are among the different conformations of A*β* aggregates found in human AD brains; these neurotoxins bind to neurons and induce synaptic loss, microglia and astrocyte activation, and Tau hyperphosphorylation. Moreover, A*β*Os reproduce most AD pathological hallmarks when injected into animal model brains [2, 3]. The cellular and molecular mechanisms by which A*β*Os perturb normal neuronal function have been extensively investigated [4]. In this regard, the excessive generation of reactive oxygen species (ROS) produced by A*β*Os represents an important source of neuronal damage [5]. The consequent redox imbalance generated by A*β*Os contributes to the pathological cascade in AD and other neurodegenerative diseases, in which oxidative stress is a common pathological feature [6, 7].

Oxidative stress occurs concomitantly with the deregulation of Ca^2+^ signaling and of downstream Ca^2+^-dependent pathways induced by A*β*Os [8, 9]; in particular, pathways downstream of N-methyl-D-aspartate (NMDA) receptors play a key role in A*β*Os induced neurotoxicity [10–12]. Aberrant activation of NMDA receptors leads to abnormal changes in gene expression that underlie A*β*Os-induced morphological and functional defects [13, 14]. The abnormal NMDA receptor-mediated Ca^2+^ signaling induced by A*β*Os activates the protein phosphatase calcineurin, leading to downstream activation of the transcription factor NFAT [15], which promotes spine loss [11].

Astaxanthin (ATX), a red-orange carotenoid that originates the pink or red color of salmon, trout, lobster, shrimp, and other sea organisms, exhibits antioxidant, anti-inflammatory, and antiapoptotic effects. Recently, ATX was shown to protect neurons in experimental models of acute injuries, chronic neurodegenerative disorders, and neurological diseases and was proposed as a beneficial strategy to treat neurological diseases [16]. Although other antioxidants, such as resveratrol, have been shown to protect neurons from injury in similar model systems [17], ATX presents many advantages compared to other antioxidants displaying protective effects. Albeit ATX has a very similar structure to that of other carotenoids such as lutein and zeaxanthin, and it has some structural differences in the arrangement of its hydroxyl groups that provide ATX with unique characteristics. Among others properties, (i) ATX has much higher antioxidant power than other members of the carotenoid family [18]; (ii) it chelates several metal ions, preventing metal ion-induced oxidative stress [19]; (iii) it has anti-inflammatory properties [18]; (iv) it crosses the blood brain barrier, allowing free access to the central nervous system [20]; (v) it acts as damper of singlet oxygen levels [21]. The combination of these properties makes ATX a very attractive candidate for use against certain diseases of the central nervous system that are caused by increases in ROS, such as superoxide anion, hydroxyl radical, and hydrogen peroxide. Consequently, ATX has been successfully used to decrease oxidative stress in elderly patients [22] and to improve neuronal function after brain ischemia [23].

Here we investigated the possible protective effects of ATX against some of the well-known deleterious effects of A*β*Os on primary hippocampal neurons. Our results strongly suggest that ATX protects neurons from the noxious effects which A*β*Os exert on mitochondrial ROS production, NFATc4 activation, and downregulation of RyR2 gene expression, suggesting that this natural antioxidant agent may represent a future approach to treat AD.

## 2. Experimental Procedures

### 2.1. Materials

A*β* peptide (A*β*1–42) was purchased from Bachem Inc. (Torrance, CA). ATX was extracted from* Lithodes antarcticus* (BIOTEX S.A., Santiago, Chile). Hexafluoro-2-propanol (HFIP) was from Merck (Darmstadt, Germany) and dimethyl sulfoxide (DMSO) from Sigma-Aldrich (St. Louis, MO). TRIzol reagent, B27 supplement, Neurobasal medium, Dulbecco's modified essential medium (DMEM), Lipofectamine 2000, and the DNA binding dye SYBR green (Platinum SYBR Green qPCR SuperMix UDG) were from Invitrogen (Carlsbad, CA). The live/dead kit was from Molecular Probes (Chicago, IL), the Ambion DNA-free*™* Kit from ThermoFisher Scientific (Chicago, IL), and the ImProm-II*™* Reverse Transcriptase kit from Promega (Madison, WI). The pEGFP-C1 NFAT3 (NFATc4) (plasmid #10961; full-length human NFATc4) was a gift from Dr. J. D. Molkentin (Cambridge, MA) [24]. The HyperMito plasmid was from Evrogen (Moscow, Russia). The amplification system (MX3000P) was from Stratagene (La Jolla, CA).

### 2.2. Preparation of A*β*Os

A*β*_1–42_ peptide, prepared as a dried hexafluoro-2-propanol (HFIP) film as described previously [9], was stored at −80°C for up to 4 months. Prior to use, this peptide film was dissolved in sufficient sterile DMSO to make a 5 mM stock solution. To prepare A*β*Os as previously described [25, 26], the 5 mM peptide solution was subsequently diluted to 100 *μ*M with cold phosphate buffered saline (PBS), aged overnight at 4°C and centrifuged at 14,000 ×g for 10 min at 4°C to remove insoluble aggregates (protofibrils and fibrils). The supernatant containing soluble A*β*Os was transferred to clean tubes and stored at 4°C. Only fresh A*β*O preparations (2 days-old maximum) were used in all experiments.

### 2.3. Primary Hippocampal Cultures

Cultures were prepared from eighteen-day-old embryos obtained from pregnant Sprague-Dawley rats as previously described [25–29]. Briefly, brains were removed and placed in a dish containing HANKS-glucose solution. Hippocampi were dissected and, after stripping away the meningeal membranes, cells were gently dissociated in HANKS-glucose solution, centrifuged, and resuspended in DMEM medium supplemented with 10% horse serum. Dissociated hippocampal cells were plated on polylysine-coated plates and after 1 h DMEM was replaced by Neurobasal medium supplemented with B-27. Cultures were incubated for 15–21 days* in vitro* (DIV) at 37°C in a humidified 5% CO_2_ atmosphere prior to experimental manipulations. The resulting cultures were highly enriched in neuronal cells, identified with neuronal anti-MAP-2, with a glial content <24% [25]. The Ethics Committee of the Faculty of Medicine, Universidad de Chile, approved the bioethical protocol of this study. All procedures were performed in accordance with the Guideline for the Care and Use of Laboratory Animals from the National Institutes of Health, USA. Animals were housed under a 12 h light/dark cycle in a temperature-controlled room at 24 ± 1°C with free access to food and water. Animals were euthanized under deep anesthesia to avoid animal suffering at each stage of the experiment.

### 2.4. Cell Viability Assay

To evaluate the effect of ATX on the cell viability of cultured hippocampal neurons maintained* in vitro* for 14 days (14 DIV), cultures were treated for 24 h with different ATX concentrations (1 nM, 10 nM, 100 nM, 1 *μ*M, 10 *μ*M, and 100 *μ*M) and cell viability was evaluated by the live/dead kit following the manufacturer's instructions as previously described [29]. Briefly, after removal of the culture, medium cells were gently washed three times with warm PBS-glucose and incubated at room temperature for 30 min in the presence of 2 *μ*M calcein AM ester and 1 *μ*M ethidium homodimer in PBS-glucose. Live neurons were identified by green calcein fluorescence and dead neurons were identified by the red fluorescence of DNA-bound ethidium. Cells were examined and counted on a Nikon® Eclipse Ti-Eat at 20x magnification. At least three random fields were imaged per culture well (three replicate wells were used per experimental condition in each experiment) and about 500 cells were counted in each well. Six independent experiments were performed with different neuronal cultures. Cell viability was expressed as percentage relative to the untreated control cultures, which exhibited 85% cell viability on average.

### 2.5. Determination of Mitochondrial Hydrogen Peroxide Generation

Cultures grown in 25 mm glass plates were transiently transfected with the HyperMito plasmid at 11–14 DIV, using a proportion of 1 : 2 DNA : Lipofectamine 2000®. HyperMito is a fusion protein of the permuted circular yellow fluorescent protein (YFP) and the regulatory domain of the transcription factor OxyR, which contains two cysteines that oxidize in response to H_2_O_2_ generation and form a disulfide bridge producing a conformational change that causes an increase in YFP fluorescence [30]. One-day after transfection, cultures incubated in Neurobasal medium supplemented with B-27 were treated for 1.5 h with 0.1 *μ*M ATX, rinsed three times with modified Tyrode solution plus 0.1 *μ*M ATX, and maintained in this solution during the experiment. At the microscope stage, cultures were stimulated with 500 nM A*β*Os and fluorescence signals from neuronal cells (identified as such by morphology) were recorded every 6 s in a Carl Zeiss LSM Pascal 5 confocal microscope system using 63x Oil DIC objective, excitation 488 nm, and argon laser beam. Changes in mitochondrial H_2_O_2_ levels are presented as *F*/*F*_0_ values, where *F* corresponds to the experimental fluorescence and *F*_0_ corresponds to the basal fluorescence.

### 2.6. Nuclear Translocation of NFATc4-eGFP

Cultures grown in 25 mm glass plates were transiently transfected with a plasmid of a fusion protein encoding a green fluorescent protein (GFP) and NFATc4 [24] at 13–15 DIV using a proportion of 1 : 2 DNA : Lipofectamine 2000®. Cultures maintained in Neurobasal medium (supplemented with B-27) were treated one day after transfection for 1.5 h with 0.1 *μ*M ATX, previous to the addition of 500 nM A*β*Os for 6 h. Neurons were then fixed with 4% paraformaldehyde, washed three times with PBS, and incubated with Hoechst for 5 minutes for nuclear staining. Covers were mounted in DAKO mounting medium for microscope observation. The subcellular localization of NFATc4-eGFP was visualized in cells using a Carl Zeiss LSM Pascal 5 laser scanning confocal with the 40x objective lens. Data were analyzed using the ImageJ software (NIH). To calculate the NFATc4 ratio of nucleus versus cytoplasm, the fluorescence intensity of nuclear NFATc4 was divided by the intensity of cytoplasmic NFATc4. Nuclear translocation of NFATc4 was determined by EGFP fluorescence intensity values from a region of interest (ROI) in the nucleus, as indicated by the overlap of EFGP staining with Hoechst nuclear staining. Background fluorescence was corrected by using a ROI devoid of cells and values were normalized to their respective areas.

### 2.7. RNA Isolation and PCR Analysis

To determine RyR2 mRNA levels, neurons were treated for 1.5 h with 0.1 *μ*M ATX prior to incubation with 500 nM A*β*Os for 6 h. To extract RNA cells were lysed as described in previous work [25]. Total RNA was isolated using TRIzol reagent. To remove any contaminating genomic DNA, a DNAase digestion step with Ambion DNA-free*™* Kit was included. RNA purity was assessed by the 260/280 absorbance ratio and RNA integrity by gel electrophoresis. cDNA was synthesized from total RNA (2 *μ*g) using the ImProm-II*™* Reverse Transcriptase kit. Twenty-five ng of cDNA was used in 20 *μ*L final volume for PCR amplification (Applied Biosystem Thermal Cycler). Amplification was performed using the primers and conditions detailed previously [25]. Real-time quantitative PCR (qPCR) was performed in an amplification system (MX3000P) using the DNA binding dye SYBR green (Brilliant III SYBER-GREEN Master Mix). Levels of RyR mRNA were calculated by the relative 2^−ΔΔCt^ method [31] and normalized with respect to levels of *β*-actin mRNA. Dissociation curves were analyzed to verify purity of products. All samples were run in triplicate.

### 2.8. Statistics

Results are expressed as mean ± SEM. The significance of differences was evaluated using Student's *t*-test for paired data and with one-way ANOVA followed by Bonferroni's* post hoc* test for multiple determinations.

## 3. Results

Previous studies showed that ATX, by attenuating oxidative damage, lipid peroxidation, and inhibiting the mitochondrial-related apoptotic pathway, protects hippocampal neurons against epilepsy-induced cellular loss [32]. Additionally, ATX prevents inflammation injury and improves cognition in diabetic mice [33]. To investigate the possible neuroprotective role of ATX against the toxic effects produced by A*β*Os, we treated primary hippocampal cultures with sublethal concentrations of A*β*Os (500 nM) in the presence or absence of ATX.

**Figure 1:**
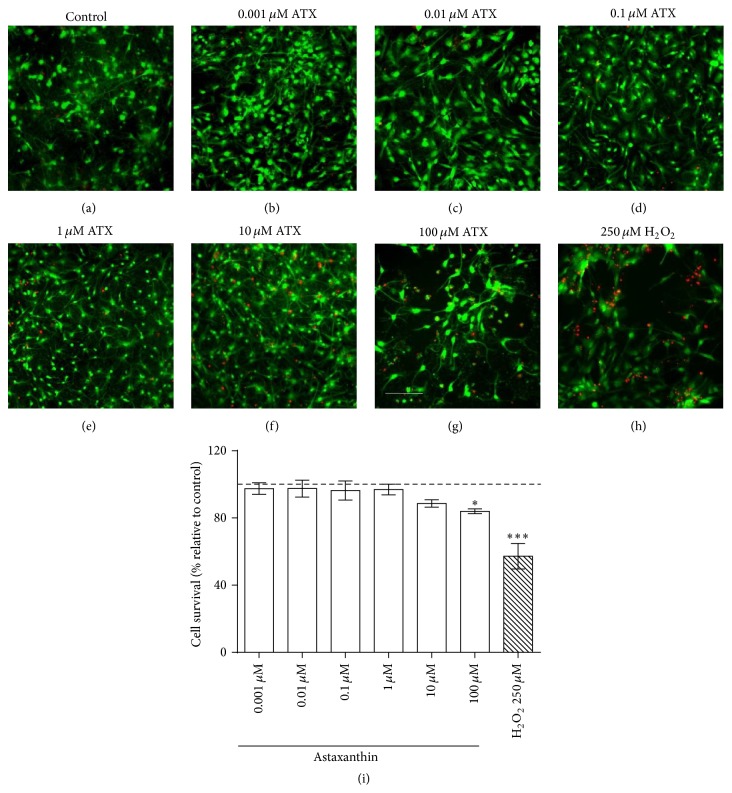
Lack of toxicity of ATX (≤10 *μ*M) to primary hippocampal cultures. Representative live/dead fluorescence images ((a)–(h)) of neuronal hippocampal cultures (13–15 DIV) incubated for 24 h in the presence of vehicle (a) or treated with 0.001 *μ*M (b), 0.01 *μ*M (c), 0.1 *μ*M (d), 1 *μ*M (e), 10 *μ*M (f), and 100 *μ*M (g) ATX. In (h), 250 *μ*M H_2_O_2_ was used to induce cell death. Live and dead neurons were identified by green calcein and red DNA-bound ethidium fluorescence, respectively. Scale bar: 50 *μ*m. (i) shows quantitative analysis of cell survival incubated with different concentrations of ATX (white bars) and under H_2_O_2_ stimulation (hatched bar). Results are expressed as percentages relative to the viability of the control, untreated cultures. Values correspond to mean ± SE of six independent experiments (*n* = 6, corresponding to cultures from 6 different animals; in all experiments, each condition was tested at least in triplicate), with different neuronal cultures. Control cultures exhibited 85% of cell viability on average. Statistically significant differences among experimental conditions were evaluated by one-way ANOVA followed by Bonferroni's multiple comparison test (^*∗*^ *p* < 0.05 and ^*∗∗∗*^ *p* < 0.0001 compared to control).

We first determined cell viability of primary hippocampal cultures (14 DIV) exposed for 24 h to different concentrations of ATX (ranging from 0.001 to 100 *μ*M). Figures 1(a)–1(h) show representative live/dead images of control cultures (a), of cultures treated with different ATX concentrations ((b)–(g)) or with 250 *μ*M H_2_O_2_ (h) to induce cell death. Live cells display calcein green fluorescence while dead cells exhibit punctuated ethidium red fluorescence.  Figure 1(i) shows the quantitative analysis of cell viability expressed as percentages of control, determined in six independent experiments performed in six neuronal cultures. Control neurons presented at least 85% cell survival. Treatment with ATX concentrations ≤10 *μ*M did not decrease cell viability when compared to control cultures, while treatment with 100 *μ*M ATX induced 20% cell death. For comparison, treatment with 250 *μ*M H_2_O_2_ elicited 40% neuronal death.

**Figure 2:**
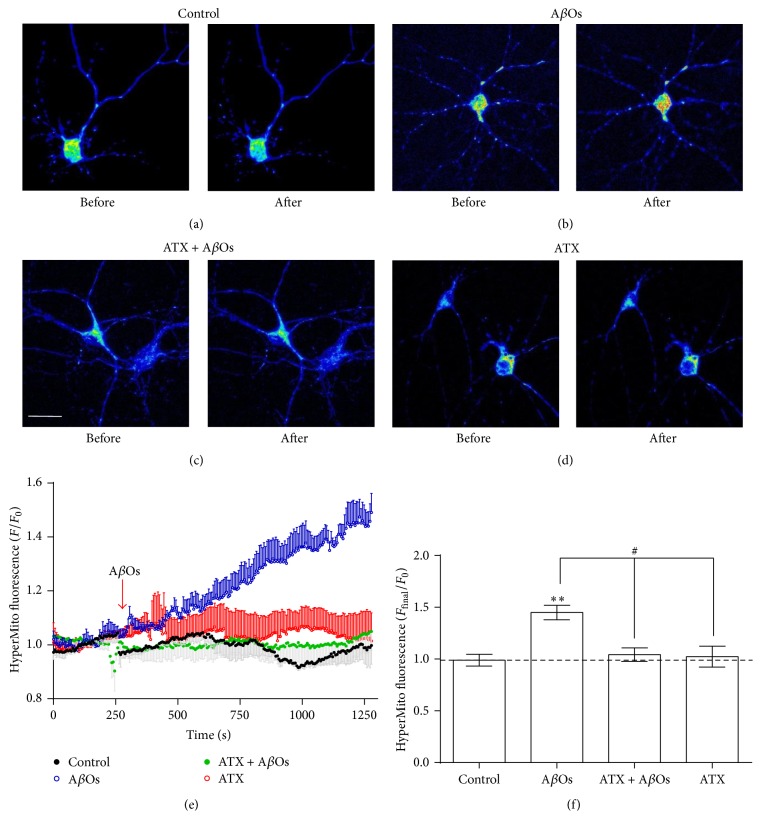
ATX prevents mitochondrial H_2_O_2_ generation induced by A*β*Os. Hippocampal neurons (13–15 DIV) were transfected with HyperMito, 24 h before the experimental maneuvers. ((a)–(d)) show representative pseudocolor images of hippocampal neurons expressing the HyperMito protein, collected before (250 s, left images) or 1000 s after (1250 s, right images) the addition of vehicle ((a) and (d)) or of 500 nM A*β*Os ((b) and (c)). Higher fluorescence intensity levels are expressed by the red color in a pseudocolor scale, while lower intensity levels are expressed by blue color. Scale bar: 10 *μ*m. (e) shows representative time courses of HyperMito fluorescence, recorded in neuronal soma after the addition of vehicle (black symbols) or 500 nM A*β*Os in the absence (blue symbols) or presence (green symbols) of 0.1 *μ*M ATX preincubated for 1.5 h, which alone did not induce changes in HyperMito fluorescence (red symbols). Fluorescence changes (mean ± SE) are expressed as (*F*/*F*_0_), where *F*_0_ corresponds to the basal fluorescence recorded in the soma before A*β*Os addition. The graph illustrates average values from 2 ROIs registered at the soma of neurons recorded in the visual field (*n* = 4). Values correspond to four different experiments performed in four cultures from four different animals; each condition was tested in duplicate. (f) shows the values of *F*/*F*_0_ obtained at the end of the experiment (time 1250 s) to each condition and bars represent mean ± SE. Statistically significant differences among experimental conditions were evaluated by one-way ANOVA followed by Bonferroni's multiple comparison test (^*∗∗*^ *p* < 0.001 compared to control; ^#^ *p* < 0.05 compared to indicated conditions).

Considerable evidence points to brain oxidative stress as an important event in the early stages of AD [5]. In particular, enhanced generation of ROS, such as H_2_O_2_ and hydroxyl radicals, has been proposed as a key molecular mechanism underlying the pathogenesis of AD [34]. Since A*β*Os induce neuronal ROS production [9], including mitochondrial ROS production [35], we tested whether ATX prevents mitochondrial H_2_O_2_ generation induced by A*β*Os. To this purpose, we transfected neurons with a plasmid that codes for the HyperMito protein, a H_2_O_2_ fluorescent sensor with mitochondrial destination; 24 h after transfection we added A*β*Os (500 nM) to the cultures and recorded fluorescence levels for 20 minutes. The representative images illustrated in Figure 2 show that control neurons (Figure 2(a)) did not display significant fluorescence changes when comparing the image taken before vehicle addition (250 s), with the final image collected at the end of the record (1250 s). Hippocampal neurons responded to A*β*Os with a significant increase in probe fluorescence, as illustrated by the representative images (Figure 2(b)), recorded before (250 s) and 1000 s after A*β*Os addition (1250 s). In contrast, neurons preincubated for 1.5 h with 0.1 *μ*M ATX and then treated with 500 nM A*β*Os (Figure 1(c)) or preincubated with ATX and vehicle (Figure 1(d)) did not exhibit significant fluorescence changes when comparing the images taken at the final and the initial time point, recorded before vehicle or A*β*Os addition. The time courses of average fluorescence changes recorded in neurons stimulated with 500 nM A*β*Os or vehicle in the presence or absence of ATX, shown in Figure 2(e), indicate that A*β*Os addition to control neurons promoted mitochondrial H_2_O_2_ production within minutes (blue trace). Neurons preincubated for 1.5 h with ATX (0.1 *μ*M) did not exhibit changes in fluorescence following addition of 500 nM A*β*Os (green trace) or of saline (red trace). The relative fluorescence (*F*_final_/*F*_0_) values of neurons maintained in different conditions, plotted in Figure 2(f), show that 500 nM A*β*Os induced a significant increase in mitochondrial H_2_O_2_ content, which was prevented by preincubation with 0.1 *μ*M ATX.

**Figure 3:**
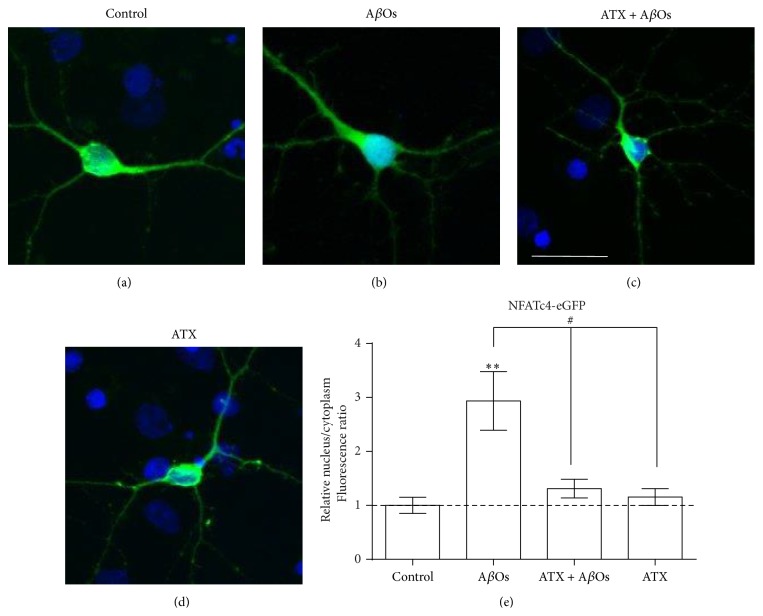
ATX prevents A*β*Os-induced NFATc4 activation. Hippocampal neurons (13–15 DIV) were transfected with EGFP-NFATc4 24 h before the experiments. ((a)–(d)) show representative images of intracellular distribution of EGFP-NFATc4 (green fluorescence) and of nuclear staining with Hoechst (blue fluorescence) in hippocampal neurons. (a) Neurons treated with vehicle, (b) stimulated with 500 nM A*β*Os for 6 h, (c) preincubated with 0.1 *μ*M ATX for 1.5 h before A*β*Os addition, or (d) incubated with ATX for 7.5 h. Scale bar: 10 *μ*m. (e) shows the quantification of four different experiments (*n* = 4) in cultures from four different animals; each condition was tested in duplicate (in total, 15–25 cells were analyzed per condition). The results are expressed as the mean ratio of nuclear/cytoplasmic fluorescence intensity ± SE, relative to control cells. Statistically significant differences among experimental conditions were evaluated by one-way ANOVA followed by Bonferroni's multiple comparison test (^*∗∗*^ *p* < 0.001 compared to control; ^#^ *p* < 0.05 compared to indicated conditions).

A requisite step for the sustained synaptic plasticity processes underlying learning and memory is an elevation in intracellular-free Ca^2+^ concentration, which plays a central role in Ca^2+^-dependent gene transcription [36–38]. Indeed, defective Ca^2+^ signaling is believed to underlie AD neuronal pathology [39]. The activation of calcineurin, which promotes the downstream stimulation of the transcriptional factor NFAT that is engaged in dendritic and axonal development, synaptogenesis, and neuronal survival [40], plays a prominent role among activity-dependent Ca^2+^-signaling pathways. In particular, the isoform NFATc4 is activated by prolonged Ca^2+^ signals [41]. Moreover, activation of NFATc4 has been demonstrated* in vitro* and* in vivo* in AD [42]. Of importance, NFATc4 activation by A*β* invokes morphological changes such as neuritic dystrophy and loss of dendritic branching and spines, effects that are prevented and reverted by inhibitors of the calcineurin/NFAT pathway [42, 43]. Also* postmortem* studies showed that, in the hippocampus of patients, activation of NFATc4 correlates with cognitive deficits [44, 45]. To investigate whether preincubation with ATX prevents the activation of NFATc4 by A*β*Os, we determined its translocation to the nucleus in hippocampal neurons transfected with plasmid codifying for the fusion protein of NFATc4 with GFP. We preincubated neurons 24 h after transfection with 0.1 *μ*M ATX for 1.5 h and exposed them to A*β*Os (500 nM) for an additional 6 h period. As illustrated in Figure 3(a), control neurons displayed GFP fluorescence mainly in the cytoplasm, indicating that in this condition NFATc4 was inactive. Incubation with A*β*Os for 6 h induced nuclear translocation of NFATc4/GFP, indicating that A*β*Os induce NFATc4 activation (Figure 3(b)). Previous incubation with ATX did not change the cytoplasmic distribution of NFATc4 in neurons incubated with A*β*Os for 6 h (Figure 3(c)) or in control neurons (Figure 3(d)). The average results from four experiments (Figure 3(e)) show that incubation of neurons with A*β*Os for 6 h increased 3-fold the nuclear/cytoplasmic ratio; this increase did not occur in neurons pretreated with ATX.

**Figure 4:**
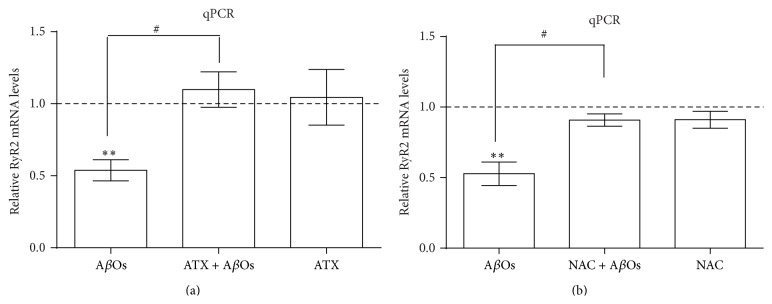
ATX prevents A*β*Os-induced RyR2 mRNA downregulation. Hippocampal cultures were preincubated with 0.1 *μ*M ATX (a) or 10 mM of NAC (b) for 1.5 h or 30 minutes, respectively, before incubation for 6 h with 500 nM A*β*Os. Relative RyR2 mRNA levels were determined with qPCR, normalized to *β*-actin mRNA levels, and expressed as fold over control. Values represent mean ± SE (*n* = 6) from experiments performed in cultures from six different animals; each condition was tested in triplicate. Statistically significant differences among experimental conditions were evaluated by one-way ANOVA followed by Bonferroni's multiple comparison test (^*∗∗*^ *p* < 0.001 compared to control; ^#^ *p* < 0.05 compared to indicated conditions).

We have previously shown that brain derived neurotrophic factor (BDNF) positively regulates the expression of intracellular RyR2 Ca^2+^ channels in hippocampal cultures [46], whereas A*β*Os downregulate RyR2 expression during synaptotoxicity [25]. In agreement with our previous results, incubation for 6 h with A*β*Os (500 nM) significantly decreased RyR2 mRNA levels to approximately 54% (Figure 4(a)). Preincubation with ATX (0.10 *μ*M) did not modify RyR2 mRNA levels but resulted in complete prevention of the reduction of RyR2 mRNA levels promoted by A*β*Os (Figure 4(a)). These results suggest a possible link between mitochondrial ROS generation and RyR2 expression. In addition, we found that the general antioxidant agent N-acetyl-L-cysteine (NAC), which is a cellular precursor of glutathione, also protects primary hippocampal neurons from the RyR2 mRNA decrease induced by A*β*Os (Figure 4(b)). Although the results presented in Figure 4 may be interpreted as an indication that RyR2 protein content was better preserved by treatment with ATX compared to NAC, these effects were not significantly different (not shown).

## 4. Discussion

Alzheimer's disease is the most common form of dementia worldwide. Among the most important risk factors for the development of AD are human conditions that have been associated with oxidative stress and chronic inflammation, which include aging, cardiovascular diseases, diabetes, hypertension, brain trauma, and high alcohol consumption [47, 48]. On the other hand, there are factors considered protective, as regular exercise and the consumption of diets rich in antioxidants [48, 49]. In the search for therapeutic strategies that could prevent AD, several attempts have been made to slow the disease progression with antioxidant agents [50]. Briefly, several studies carried out in* in vitro* and* in vivo* models of AD have shown some positive results of antioxidants. The main mechanisms proposed to explain these effects are (1) mimicking endogenous catalytic enzymes (mainly superoxide dismutases (SOD), catalases (CAT)) [51] and metabolic precursors of endogenous antioxidants system (Glutathione) [52]; (2) acting like ROS scavenger; and (3) causing SIRT activation [53]. Although antioxidants have been widely studied as an alternative strategy to prevent or treat AD, direct evidence is still required to support their use in the treatment of patients suffering from AD [54, 55].

Antioxidant agents are distinct chemical entities with structures that command their different modes of action; this structural diversity imparts each antioxidant agent with a unique biochemical profile, which is reflected in different sites of action and biological activities. ATX is classified as a lipophilic antioxidant of the carotenoid family, and its main protective mechanisms rely on its capacity to act as a singlet oxygen quencher and free radical scavenger. However, after scavenging reactive-free radicals, ATX is transformed into a carotenyl radical by hydrogen abstraction; this process can lead to a switch from a beneficial antioxidant agent to a damaging prooxidative one, which could explain ATX toxicity when used at higher concentrations (100 *μ*M) [56]. Here, we studied the protective properties of ATX against the noxious effects of A*β*Os on primary hippocampal cultures and compared some of its effects with the protective actions of NAC. We also compared the effects of NAC because this is a classical and very widely studied antioxidant. Although NAC also acts as a ROS scavenger, its principal action stems from its role as a precursor of cysteine, the rate-limiting factor in the* de novo* synthesis of glutathione (GSH). In this sense, contrary to ATX, NAC is an indirect antioxidant, which, as a precursor of the antioxidant GSH, has a safer toxicity profile, allowing the use of higher concentrations [57]. Both antioxidant agents have been studied in the context of central nervous system disorders [16, 58], but evidence related to NAC is more abundant.

We have previously shown that incubation of hippocampal neurons* in vitro* with NAC, a glutathione precursor molecule with antioxidant properties, completely prevents the aberrant increase in intracellular Ca^2+^ levels and the mitochondrial fragmentation induced A*β*Os [25, 52]. In previous work from our laboratory, we have also shown that reducing agents suppress RyR activation by Ca^2+^ in cortical neurons [59]. Also the administration of NAC through drinking water to a transgenic mouse model of AD (mouse APP/PS-1) suppressed the protein oxidation and nitrosylation in the brains of mice aged 9 and 12 months [60]. The above results are consistent with the idea that NAC modulates the activity of RyR by avoiding the aberrant intracellular ROS increase and hence the enhanced Ca^2+^ release produced by ROS-modified RyR, induced by A*β*Os.

Astaxanthin is a natural carotenoid product that is used in nutritional supplements, which can be extracted from* Lithodes antarcticus*. ATX has been shown to quench singlet oxygen and to scavenge free radicals [61]; ATX antioxidant properties reside in its polar ionic rings and nonpolar conjugated carbon–carbon bonds and are 10-fold greater than those of other carotenoids [62]. In addition to the ROS scavenging properties attributed to ATX, several studies have shown that ATX, alone or in combination with omega-3 fatty acids, protects cells by inducing antioxidant activity via the nuclear factor (erythroid-derived 2)-like 2 (Nrf2) Nrf2/heme oxygenase-1 (HO-1) signaling pathway [63–65]. Several studies have implicated Nrf2 in the induction of HO-1, which is the enzyme that catalyzes the first and rate-limiting step of heme metabolism [66, 67]. HO-1 activity protects tissue during inflammatory stress in various conditions through the degradation of prooxidant heme and the production of carbon monoxide (CO) and bilirubin, both of which have anti-inflammatory and antiapoptotic properties, especially in ROS-dependent perturbations associated with metabolic syndrome [67].

In this work, we tested the possible neuroprotective effects of ATX on some of the noxious effects induced by A*β*Os on primary hippocampal cultures. We found that ATX prevented the mitochondrial generation of H_2_O_2_, the nuclear translocation of NFATC4, and the decrease of RyR2 mRNA levels induced by A*β*Os. These protective effects may result from the reduction of intracellular ROS promoted by ATX. Although ROS have important roles in cell signaling and normal neuronal function, excessive ROS generation, such as that produced by A*β*Os, has deleterious effects on neuronal function, which include the significant damage to DNA, RNA, proteins, and polyunsaturated fatty acids in lipids caused by irreversible oxidation. Normally, cells defend themselves against ROS damage through intracellular and extracellular defenses, in particular through enzymes such as SOD, CAT, lactoperoxidases, and glutathione peroxidases. ATX supplementation not only lowers ROS levels but also leads to an important functional recovery of the antioxidant network [68], including SOD, which catalyzes the dismutation of superoxide anion to O_2_ and H_2_O_2_, and CAT, which protects cells from oxidative damage by catalyzing the decomposition of H_2_O_2_ to water and O_2_.

Recent clinical studies showed that ATX promotes significant reductions in cardiovascular risk markers of oxidative stress and inflammation [69]; ATX also has considerable potential for both the prevention and treatment of various chronic inflammatory disorders, such as cancer, asthma, rheumatoid arthritis, metabolic syndrome, diabetes, and diabetic nephropathy, as well as gastrointestinal, hepatic, and neurodegenerative diseases [56]. In rats, ATX supplementation in the diet for four weeks markedly decreases the level of malondialdehyde (MDA), nitric oxide, and advanced protein oxidation products in the cortex, striatum, hypothalamus, hippocampus, and cerebellum [68]. Also ATX increases the activity of CAT and SOD enzymes as well as the level of glutathione in the brain [68]. Additionally, ATX exhibits protective effects against the neurotoxicity induced by Abeta_25–35_ peptide aggregates in PC12 and neuroblastoma (SH-SY5Y) cells [70, 71]. These results are in agreement with the main conclusions presented in this work.

Besides the role of ROS and oxidative stress in AD, there are many studies linking this disease with a sustained increase in intracellular Ca^2+^ levels [39, 72, 73]. We have previously reported that A*β*Os addition to primary hippocampal neurons causes an increase in Ca^2+^ entry to the cytoplasm via NMDA receptors, which promotes RyR-mediated Ca^2+^ release [25]. We also showed that previous incubation of primary neurons with NAC prevents the emergence of sustained Ca^2+^ signals induced by A*β*Os [52]. These findings emphasize the participation of ROS in the maintenance of the Ca^2+^ signals induced by A*β*Os. On the other hand, intracellular Ca^2+^ chelators such as BAPTA-AM prevent the ROS generation induced by A*β*Os, indicating that there is a crosstalk between the ROS and Ca^2+^ signals induced by A*β*Os [9]. In this context, the RyR channel appears as an important actor since its activity and expression are regulated by this crosstalk [74]; thus, RyR channels act as coincident detectors of Ca^2+^ and ROS due to the presence of cysteine residues that are reversible modified by oxidants, enhancing RyR activation by Ca^2+^ [75]. We showed that incubation of primary hippocampal neurons with A*β*Os causes an important downregulation of RyR2 mRNA and protein contents and proposed that these reductions are crucial to the synaptotoxicity induced by A*β*Os [25]. Of note,* postmortem* samples of patients who died with AD display significantly reduced RyR2 expression at early stages of the disease [76].

Dysregulation of Ca^2+^-dependent gene transcription plays a critical role in synaptic plasticity and memory defects [77]. A*β*Os induce calcineurin activation, which leads in turn to activation of its canonical target, the transcriptional factor NFAT. The damage observed in the cortex and hippocampus of* postmortem* AD patients during the progression of the disease, correlates with activation of the calcineurin/NFAT pathway in both glial and neuronal cells [44, 45]. Activation of this pathway, even in the absence of A*β*, is sufficient to produce a virtual phenocopy of A*β*-induced dystrophic neurites, dendritic simplification, and dendritic spine loss in both neurons in culture and in adult mouse brain [42]. Thus, A*β*Os appear to mediate the neurodegeneration of AD, at least in part, through calcineurin activation and subsequent stimulation of NFAT-mediated downstream cascades.

Calcineurin is susceptible to significant (up to 15 times) and reversible activation by Ca^2+^/CaM. This activation is favored during chronic elevations of Ca^2+^ in the cytoplasm resulting from ER stress caused by exposure to misfolded proteins [78]. However, calcineurin activity is also redox-sensitive, so that oxidation of calcineurin strongly inhibits its phosphatase activity. Some possible mechanisms to explain this apparent paradox, that is, increased activity in conditions of increased ROS generation, have been discussed in studies from other groups [78, 79]. Cleaved forms of the enzyme, which were found in AD brains [79], are constitutively active.

**Figure 5:**
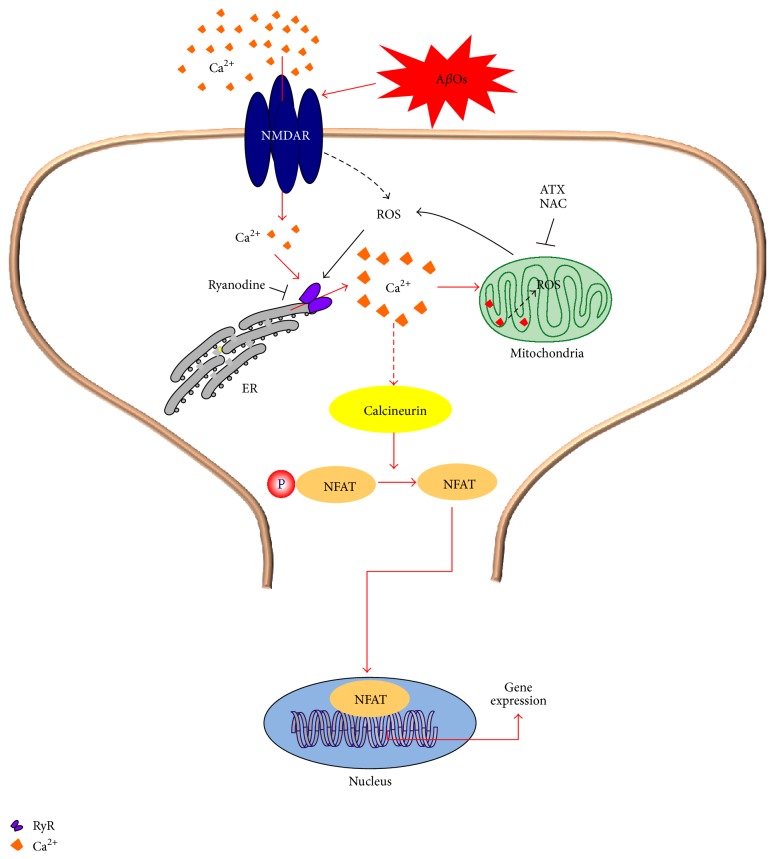
Scheme showing a possible mechanism to explain ATX neuroprotection over the deleterious effects of A*β*Os. A*β*Os promote increased ROS generation and induce abnormal Ca^2+^ signals in primary hippocampal neurons, which arise initially from Ca^2+^ entry through NMDA receptors; these entry signals are subsequently amplified by Ca^2+^ release through RyR channels costimulated by Ca^2+^ and the increased ROS levels generated response to A*β*Os [25]. Activation of RyR-mediated Ca^2+^ release by ROS [59, 77] induces mitochondrial Ca^2+^-uptake, which is prevented by ryanodine at inhibitory concentrations [27]. These abnormal cytoplasmic Ca^+2^ signals promote NFATc4 translocation, which induces deleterious changes in gene expression and dendritic spine morphology [42]. ATX and NAC, either by scavenging ROS/RNS or by increasing antioxidant defenses, would prevent abnormal A*β*Os induced RyR-mediated Ca^2+^-induced Ca^2+^ release and thus would prevent the harmful effects of enhanced NFAT nuclear translocation.

Here, we demonstrated for the first time that ATX inhibits the nuclear translocation of NFAT induced by A*β*Os, suggesting that the calcineurin/NFAT pathway responds to the increased neuronal oxidative tonus induced by A*β*Os. Additionally, we show that ATX inhibits the downregulation of RyR2 mRNA levels promoted by A*β*Os. These results suggest that excessive ROS decrease RyR2 expression, although it is not known if the calcineurin/NFAT pathway mediates this decrease. Previous work indicates that the RyR2 protein plays an important role in hippocampal synaptic plasticity processes [77], so its downregulation by A*β*Os may also contribute to their synaptotoxic effects. We previously showed that incubation of primary hippocampal neurons with A*β*Os for a period of 6 h prevents the rapid spine remodeling prompted by caffeine-induced RyR-mediated Ca^2+^ release or by BDNF, which also requires RyR-mediated Ca^2+^ signals; these results suggest that the RyR2 decrease induced by A*β*Os produces a significant reduction of RyR2-mediated Ca^2+^ signals in response to BDNF, leading to defective synaptic remodeling [25]. Thus, decreased RyR2 protein expression may contribute to impair synaptic plasticity in AD (Figure 5).

The present results indicate that ATX, via its antioxidant properties, may prevent important deleterious effects of A*β*Os on gene expression, which might be controlled at least in part by the calcineurin/NFAT pathway. Taken together, our results demonstrate the potential of ATX to prevent synaptotoxic effects of A*β*Os in an* in vitro* model of AD. Given the neuroprotective effects of ATX against different neurological disorders, the results presented here support the idea that daily consumption of ATX may be a beneficial strategy in human health management of AD and possibly of other neurological disorders as well.
